# Acute myeloid leukemia in a 38-year-old hemodialyzed patient with von Hippel-Lindau disease

**DOI:** 10.1186/1897-4287-11-11

**Published:** 2013-08-22

**Authors:** Katarzyna Labno-Kirszniok, Teresa Nieszporek, Andrzej Wiecek, Grzegorz Helbig, Jan Lubinski

**Affiliations:** 1 Department of Nephrology, Endocrinology and Metabolic Diseases, Medical University of Silesia, Francuska Street 20/24, Katowice 40-027, Poland; 2 Department of Hematology and Bone Marrow Transplantation, Medical University of Silesia, Reymonta Street 8, Katowice 40-029, Poland; 3 Department of Genetics and Pathology, Pomeranian Medical University, International Hereditary Cancer Center, Połabska Street 4, Szczecin 70-115, Poland

## Abstract

Von Hippel-Lindau disease (VHL disease) is a hereditary cancer predisposition syndrome caused by mutations of the von Hippel-Lindau tumor suppressor gene. The gene product, pVHL, regulates the level of proteins that play a central role in protecting cells against hypoxia. Clinical hallmarks of von Hippel-Lindau disease are the development of central nervous system hemangioblastomas, renal cell carcinoma, pheochromocytoma, neuroendocrine tumors and endolymphatic sac tumors.

In this article the case of a 38-year old hemodialyzed patient who became ill with acute myeloid leukemia (AML) three years after being diagnosed with von Hippel-Lindau disease is presented.

After cytostatic treatment the patient went into complete hematologic remission but there was still residual disease at the genetic level. After consolidation therapy patient developed bone marrow aplasia and severe pneumonia. Despite intensive treatment the patient died from acute respiratory failure.

In this paper we present for the first time a case of von Hippel-Lindau disease associated with acute myeloid leukemia. No evidence of relationship between VHL disease and blood cancers has been demonstrated so far. Despite the fact that there is an increased risk of cancer development in hemodialyzed patients, cancer is a relatively rare cause of death in the dialysed population, and the most common malignancies are genitourinary cancers. It seems likely that development of acute myeloid leukemia in patient with VHL disease can be related to epigenetic alterations of the VHL gene, but further studies are needed.

## Introduction

Von Hippel-Lindau disease is a rare multiorgan autosomal dominant genetic disorder, with predilection to develop characteristic retinal and central nervous system (CNS) hemangioblastomas, renal cell carcinomas (RCCs), pheochromocytomas, endolymphatic sac tumors and pancreatic cysts [1,2] (Table 1). The incidence of von Hippel-Lindau disease is estimated to be 1 in 36,000 individuals [3]. The disease is caused by mutations of the tumor suppressor gene on chromosome 3p25, which encodes a protein VHL [4]. Constitutional mutation of the VHL gene is usually. About 20 percent cases of VHL disease result from the development of a new” mutation in the VHL gene (spontaneous mutation) and the family histories are negative [5]. Tumors associated with VHL are multifocal, bilateral and of an early onset. The average age of clinical diagnosis of retinal hemangioblastomas is 25 years, of pheochromocytomas and cerebellar hemangioma is 30 years, of renal cell carcinoma is 35–40 years [6,7]. Complete surgical removal of the solid tumors is the treatment of choice. Early detection of tumors can provide effective treatment, alleviates the disease process and prolong the survivability of patients with VHL disease [8]. The most common causes of death are complications from cerebellar hemangioblastoma (47, 7%) with an average age of death is 41 years [7,8].

**Table 1:** **Cancer incidence and types of tumors in VHL disease**[6]

**Type of cancer**	**Cancer incidence**
CNS	
Hemangioblastomas of the retina	25-60%
Endolymphatic Sac Tumors	10%
Hemangioblastomas of	
Cerebellum	44-72%
Brain stem	10-25%
Spinal cord	13-50%
Nerve roots L/S	< 1%
Supratentorial region	< 1%
Organs	
Renal cel carcinoma	25-60%
Pheochromocytoma	10-20%
Pancreatic tumors	35-70%
Epididymal cystadenomas	25-60%

This paper reports the case of a 38-year-old male with von Hippel-Lindau disease and chronically hemodialyzed due to bilateral nephrectomy for renal cell carcinoma who also became ill with acute myeloid leukemia (AML). No case of acute myeloid leukemia in hemodialyzed patient with VHL disease has been reported so far.

## Case report

We observed 38-year-old hemodialyzed male patient diagnosed with von Hippel-Lindau disease in 2008 who was admitted to the Department of Nephrology, Endocrinology and Metabolic Diseases, Medical University of Silesia in Katowice with significant anemia, increased number of leukocytes, pneumonia and with suspected endocarditis.

In June 2007 the patient underwent right nephrectomy for renal cell carcinoma and then in October 2007 he had surgery to remove a tumor from his brainstem (medulla oblongata) at the level of the posterior portion of the foramen magnum. Histological examination found hemangioblastoma which might have occurred as a feature of VHL disease.

In 2008 the Department of Genetics and Pathology, Pomeranian Medical University in Szczecin detected a constitutional mutation 407 C/T (F136S) within the VHL gene. Study of the patient’s family history showed that his father, uncle and grandmother were diagnosed with renal cell cancer. The patient underwent annual screening program in order to identify cancer manifestations early.

In October 2010, he was diagnosed with a solitary kidney tumor and qualified for surgical treatment. Histological examinations showed the tumor to be renal cell carcinoma. Postoperative course was complicated by massive hematuria leading to urgent nephrectomy resulting in chronic hemodialysis.

In March 2011, he was diagnosed with dilated cardiomyopathy and complex valves defect. In May 2011, severe central sleep apnea was determined.

In July 2011, patient was admitted to the hospital again. Blood tests showed anemia (HGB = 5, 6 g/dl), leukocytosis (WBC = 13×10^9^/l) and moderate thrombocytopenia ( PLT = 72×10^9^/l). There was no peripheral blood smear done.

Gastroscopy revealed erosive gastritis. He required blood transfusion during the hospitalization. Following tests reported increasing number of white blood cells (up to 55×10^9^/l) and reducing number of plates despite use of intravenous corticosteroids. A preliminary diagnosis was: infective endocarditis, pneumonia, erosive gastritis and duodenitis accompanied by anemia, leukocytosis and thrombocytopenia.

The patient was then transferred to the Department of Hematology and Bone Marrow Transplantation, Medical University of Silesia in Katowice. Physical examination revealed a heart murmur and splenomegaly. Blood test showed persistent anemia, decreased plateled count and hyperleukocytosis. A peripheral blood smear determined the presence of blasts.

Ultrasound imaging of the abdomen revealed enlarged spleen (14.6x9.9 cm). Bone marrow aspirate confirmed the findings of blood smear and showed the presence more than 50% of myelomonoblastic cells. The flow cytometric immunophenotyping report indicated the malignant cells were positive for HLA-DR, MPO, CD13, CD15, CD33, CD116 and cytogenetic analysis showed the inv (16) (p13;q22). Molecular analysis of bone marrow cells using RT-PCR identified a CBFB-MYH11 fusion protein.

Low cytogenetic risk patient was treated with individualized induction therapy DA: daunorubicin (60 mg/m^2^ on days 1–3) and cytosine arabinoside (200 mg/m^2^ on days 1–5). Hemodiadialyzotherapy was continued. His chemiotherapy- induced bone marrow aplasia with granulocytopenia was complicated by pneumonia.

The patient achieved a complete hematologic remission in peripheral blood, but not molecular remission after induction chemotherapy (positive RT-PCR assays for oncogene CBFB-MYH11). The Patient then received consolidation treatment with HAM (mitoxantrone 10 mg/m^2^ on days 1–2 and cytosine arabinoside 1500 mg/m^2^ on days 1–5). During the period of profound aplasia an additional hemodialysis due to hyperkalemia was required. Prolonged bone marrow aplasia resulted in severe interstitial pneumonia and septicemia. Despite intensive therapeutic measures the patient died from respiratory failure.

The autopsy revealed a pulmonary edema, massive hyperemia of the left lower lobe with pneumonia confluens.

## Discussion

In a normal oxygen environment, VHL protein plays a crucial role in degradation of specific HIF proteins (Hypoxia Inducible Factor) maintaining very low levels of HIF alpha in the cell and decreases expression of angiogenesis factors such as vascular endothelial growth factor (VEGF), platelet-derived growth factor (PDGF), transforming growth factor alpha (TGFα) and erythropoietin [9,10]. Mutations of the VHL gene disrupt the regulation of HIF resulting in overproduction of growth factors and inappropriate activation of angiogenesis in VHL-associated tumors [11]. Table 1 presents cancer incidence and types of tumors in VHL disease.

VHL-associated tumors are highly vascularized and can overproduce angiogenic peptide VEGF.

Many other HIF-independent functions of VHL have been identified [12,13]. Furthermore, studies revealed that pVHL interacts with other proteins, which are destined for degradation through the ubiquitin-proteasome proteolytic pathway. Some of these proteins include atypical protein kinase C [14], VHL-interacting deubiquitinating enzyme 1 and 2 (VDU 1 and VDU 2) [15], hyperphosphorylated large subunit (Rpb1) of RNA polymerase II [16]. Moreover, pVHL stabilizes protein Jade-1 [17] that is most highly expressed in kidney and inhibits renal cancer cell growth [18]. VHL gene product plays significant role in different processes, including regulation of metabolic genes expression, angiogenesis, erythropoiesis, apoptosis and proliferation. This wide spectrum of cell-specific functions raise some intriguing conjecture about a potential pathogenic role of VHL gene mutation in leukemia. HIF-independent function of pVHL is therefore of particular importance [12,13].

A mentioned protein Jade-1 plays an important role in promoting apoptosis [18]. VHL - interacting Jade-1 is crucial for the proper functioning of a Jade-1protein. The PHD finger–containing region (also called plant homeodomains PHD), specific DNA-binding motifs, has been shown to have a role in Jade-1 stabilization. A mutation or loss of functioning PHD domains are the pathogenic event in several human diseases, particularly in the acute leukemias [19-22].

Mutations of the VHL gene result in destabilization of Jade-1 protein which can have a fundamental impact both on clinical manifestations of von Hippel-Lindau disease and pathogenesis of renal cell carcinoma [23]. Many proteins associated with leukemia contain PHD domains such as AF10 whether MLL [24]. Moreover, leukemic fusion proteins (MLL-AF10), which arise from chromosomal translocations, do not have PHD domains. MLL-AF10 fusion protein, as a result of genetic defects, is generated in acute leukemias and is responsible for the leukemogenic process [25,26].

PHD domains are therefore important, structurally distinct parts of the proteins and prevent cancer initiation.

Knowing that stabilization of Jade-1 protein by pVHL occurs only through intact plant homeodomains, it may be assumed pVHL to have similar function to other interesting proteins with zinc finger domains [23].

Possibly the mutations in VHL gene and loss of regulatory function to competent PHD-proteins, with subsequent transcription process activation and cell-division stimulation, are involved in the pathogenesis of not only renal cell carcinoma in VHL disease but many other cancers including acute myeloid leukemia. It cannot be excluded that protein products of other defective genes have the effect of modifying the VHL protein which can play an important role in the pathogenesis of AML.

One of the reasons for the increased cancer risk among dialysis patients is the previous treatment with immunosuppressive drugs for the management of glomerulonephritis [27]. These disorders are the common causes of end stage kidney disease. It is worth noting that the patient had never received immunosuppressive therapy, chemotherapy and radiotherapy that could have induced myelodysplasia (MDS) and secondary leukemia.

It should be further considered if a relationship exists between hypermethylation in CpG islands of VHL gene and hematologic cancer development. In a study an assessment of the VHL gene methylation level have been carried out between two groups: one of 58 patients not previously treated for acute myeloid leukemia and MDS and another of 10 healthy volunteers. This study showed that with highly sensitive diagnostic method epigenetic changes were detected in the CpG Island regions in some patients with AML and MDS compared to the reference group. Because of small study population it is difficult to say if observed epigenetic changes influence VHL gene expression and clinical phenotypes of von Hippel-Lindau disease [28].

In addition, recent studies implicate that particular epigenetic regulations of some genes, including VHL gene, may play a crucial role in other hematological and non-hematological malignancies. Namely, it was demonstrated that epigenetic changes were found to be involved in pathogenesis of chronic lymphocytic leukemia [29] and endometrial cancer [30]. Moreover, these findings may have an important clinical implication in the era of hypomethylating therapy.

While analysing the clinical course of the patient, the oncologycal issues shall be considered in renal replacement therapy. Chemotherapy and its complications are a crucial therapeutic problem for this group of patients.

There is a significant immune system deficiency both inherited (white blood cells inflammatory response ability) and acquired (antibody production and immunological memory) in patients treated with repeated dialysis. Long-term dializotherapy can be considered an immunosuppressed state because of the retention of uremic toxins making patients more susceptible to infection and cancer development [31-34].

Carcinogenic risk factors associated directly with renal replacement therapy should be taken into consideration. Continuous exposure to foreign antigens as a consequence of extracorporeal blood circulation, using dialysis catheter and exposition of the peritoneal membrane to the dialysis fluids can lead to the activation of the immune system and an increase incidence rate of cancers, augumented by persistent inflamatory state [35].

Chronic hemodialysed patients have high cumulative radiation exposure because of frequent radiographic procedures [36]. A single dose of 10 mSv produces a lifetime risk of developing a solid cancer or leukemia in 1 out of 1000 cases [37].

One of the large cooperative study focused on the kidney, gallbladder, thyroid and urogenital cancers showed increased incidence of malignancies in hemodialyzed patients versus a healthy population [38,39].

Viral etiology was confirmed in pathology of some of the human cancers (e.g. human papillomavirus and cervical cancer) which suggests that inappropriate immune response in dialysed patients predisposes them to the oncogenic virus-associated neoplasia. Furthermore secondary immunodeficiency in uremic patients is far more likely to induce a poor vaccine response and ineffective passive immunization [34].

It is assessed that despite a life expectancy of dialysed patients is shorter than the development time of neoplasia, cancer risk is 4 times higher than in the general population [40]. Malignancy is a relatively rare cause of death in dialysed patients [41]. Table 2 presents the relative incidence of the most common cancers among dialysed patients.

**Table 2:** **Cancer types and incidence in end stage renal disease**[38]

		
**1.**	Lung	15,7%
**2.**	Intestinal	12,9%
**3.**	Prostate	9,9%
**4.**	Kidney	8,2%
**5.**	Breast	7,8%
**6.**	Bladder	6,6%
**7.**	Multiple myeloma	5,8%
**8.**	Non-Hodgkin lymphoma	3,4%
**9.**	Stomach	3,1%
**10.**	Leukemia	2,8%
**11.**	Cervix of uterus	1,8%
**12.**	Body of uterus	1,7%
**13.**	Liver	1,4%

There are few clinical reports about effectiveness of cancer therapy in dialysed patients, but it certainly requires a good cooperation among the team members including nephrologist, oncologist and dialysis station workers.

The most commonly used treatment is a standard chemotherapy. However, clearance of a particular drugs by dialysis, appropriate dosage adjustment and time of administration must be taken into account to avoid overdosage and related side-effects [42,43].

Hematologic malignancies are relatively rare in the dialysed population due to significant iron deficiency. Guidelines for treatment of leukemia in this group of patients have not yet been established. Chemotherapy dosage should be modified as necessary to accommodate patient’s tolerance to treatment [44-46]. It’s worth mentioning that chemotherapy for cancers susceptible to cytostatic therapy, including leukemias, increases the risk of acute tumor lysis syndrome combined with impaired renal function creates diagnostic and therapeutic difficulties [47].

## Summary

Von Hippel-Lindau disease is a rare genetic disorder characterized by hemangioblastomas, renal cell carcinoma, pheochromocytomas, neuroendocrine and endolymphatic sac tumors. There has not been found any susceptibility to hematologic cancers in VHL disease.

This is the first time that coexistence of VHL disease and leukemia has been described in available literature.

End stage renal disease and repeated hemodialysis treatment are the risk factors predisposing to cancer. Chemotherapy in patients undergoing hemodialysis is a constant challenge for the medical team consists of different specialists. Individual patient care is undoubtedly needed, with close cooperation of specialists in clinical oncology, nephrology, surgery and the medical staff of the dialysis unit.

## Competing interests

The authors declare that they have no competing interests.

## Authors’ contributions

AW Head of the Department of Nephrology, Endocrinology and Metabolic Diseases in Katowice. He coordinated the group of physicians and provided scientific guidance and reviewed the manuscript. KL followed the patient, was in charge of the therapeutic process and drafted the manuscript. TN was supervising the therapeutic process and reviewed the manuscript. GH provided hematological expertise on the case and drafted the hematological paragraphs of the manuscript. JL diagnosed the mutation in the VHL gene in the patient subject of this study and reviewed the manuscript. All authors read and approved the final manuscript.
